# Beyond pain privacy and pain meters: a new vision for pain biomarkers

**DOI:** 10.3389/fpain.2024.1397645

**Published:** 2024-10-03

**Authors:** Charles Djordjevic, Carl Y. Saab

**Affiliations:** ^1^ Nursing, Cleveland Clinic Foundation, Cleveland, OH, United States; ^2^ Department of Humanities, Lorain County Community College, Lorain, OH, United States; ^3^ Department of Biomedical Engineering, Cleveland Clinic Foundation, Cleveland, OH, United States; ^4^ School of Medicine, Case Western Reserve University, Cleveland, OH, United States; ^5^ Department of Engineering, Brown University, Providence, RI, United States

**Keywords:** pain, neurophilosophy, predictive coding, biomarker, Descartes

## Abstract

To an individual, pain is unambiguously real. To a caregiver, assessing pain in others is a challenging process shrouded in doubt. To explain this challenge, many assume that pain “belongs” exclusively to the bearer of that experience and accept the dogma that pain is private. However, privacy also entails that it is *not* possible to identify, share, or communicate that experience with others. Obviously, this is not true and the consequences of pain privacy would be devastating for healthcare. Pain is indeed unique and subjective, but not necessarily private. Pain is in fact readily communicable, though perhaps not as effectively and reliably as caregivers would like. On the other hand, healthcare systems mandate objective metrics in pain diagnosis. Smiley face caricatures are a staple of clinical practice and a universal standard for reporting pain levels. These conditions create a double paradox: Assess a private experience that is inaccessible, *and* use numerical scales to measure subjective attributes. Navigating this stressful environment, medical professionals experience intellectual dissonance, patients are frustrated, and value-based care is undermined. Offering a way out, first, we refute the privacy and objectification of pain citing philosophical, behavioral, and neuroscientific arguments. We discuss Wittgensteinian views against privacy, explore the clear evolutionary advantage of communicating pain to others, and identify neural circuits in the mammalian brain that contribute to empathy. Second, we highlight the subjectivity of pain, embracing the complexity and uniqueness of an individual's pain. We also provide compelling evidence for brain mechanisms that actively shape the pain experience according to predictive coding principles. Third, we offer a vision for the development of biomarker technologies that assess pain fairly without engendering bias against the patient's narrative. Our recommendations are based on the overwhelming appreciation that “medicine by emoji” is inadequate for capturing the multidimensional nature of pain. Our view is that the most promising candidates for pain biomarkers consist of self-reports as ground truth *augmented* by physiological signatures of biological relevance to pain. Integration of subjective and objective multimodal features will be key for the development of comprehensive pain assessment models.

## 1 Synopsis

The clinical management of pain is a significant refractory problem across healthcare systems. These difficulties are compounded by the current state of pain assessment, reliant as it is on pain scales and a “medicine by emoji” approach. In response to this, clinicians, funding agencies, and scientists are searching for reliable tools to diagnose pain. One such approach focuses on identifying and measuring biomarkers, empirical phenomena that hold out the hope of enabling healthcare providers to objectively diagnose pain and respond to it effectively with individualized treatments. Although we broadly support attempts to expand scientific research into pain biomarkers, we fear that deeply rooted philosophical confusions concerning the ground truth of pain will both stymie this vital research into biomarkers and erode value-based care. We argue that properly integrating biomarkers into pain assessment requires repudiating the contention that pain is private, roughly that only the experiencer knows for herself what pain is and what “pain” refers to [e.g., (1, 2)]. Specifically, we reject the assumption that the subjectivity of pain equates with privacy. We then draw on cutting-edge neuroscientific research, broadly Bayesian statistics, and philosophy to proffer a radically different framework for understanding pain as subjective but *not* private. Finally, we briefly discuss how this framework seamlessly integrates candidate biomarkers, artificial intelligence (A.I.)-based algorithms, etc., into pain assessment tools without cutting the patient out of the loop.

To make this case, the paper divides into a critical part which argues that both a “private” and an “objective” view of pain are equally untenable and a constructive part which rejects these presuppositions and proffers a novel account of pain, one that avoids these challenges and conceptualizes pain, its assessment and management in radically different terms.

Section 1.1 sets the stage and reminds the reader both of how important pain is for patients and how badly healthcare has failed at dealing with it effectively. Section 1.2 begins the critical part of the paper where we define “pain privacy” and discuss how and why it became ubiquitous in both the clinical and research literature. Section 1.3 proffers both neuroscientific evidence and philosophical arguments to show that this view is untenable. Section 1.4 presents the “objective” view of pain, introduces the concept of biomarkers, and discusses attempts to use them to circumvent the bottleneck of privacy, effectively cutting the patient out of the pain assessment loop. Section 1.5 argues that such a reductive use of biomarkers generates a host of problems. Section 1.6 draws from the work of Ludwig Wittgenstein, a notable philosopher and logician, and argues that both the private and the objectifying views rest on a “picture” of language that forces mutually exclusive, though dialectically reinforcing, views of pain as either Cartesian ghost or machine. In Section 1.7, we begin the constructive portion of our paper, where we reconceptualize biomarkers as a novel, and potentially vital, source of empirical evidence that can complement and supplement, *but not replace*, the ground truth of pain in a patient's narrative. Section 1.8 nests this account of biomarkers in a broader discussion of pain's subjectivity and intersubjective recognition. Section 1.9 sums up this positive proposal, and the last section discusses plausible paths forward, should our thesis prove persuasive.

### 1.1 Lack of comprehensive pain assessment tools underscores our inability to deal with pain

Pain is the number one reason for seeking medical care in the USA. Chronic pain affects a third of the US population with healthcare costs exceeding $600 billion per year. An estimated 50–100 million adults in the USA live with chronic pain that can substantially restrict work, social, and self-care activities (3). The increased prevalence of chronic pain has been associated with a substantial rise in the use of prescription opioids to treat non-cancer chronic pain, with 275 million people worldwide using opioids in 2016 and 27 million of these people developing opioid use disorders (4). As a result, more than 90 individuals in the USA die per day from opioid overdose (5). Worse, the clinical problem of pain has stubbornly persisted despite years of attempted reforms at all levels. Major revisions to quality indexing, a resurgent focus in healthcare education on pain assessment, and Congress proclaiming a “decade of pain research” have not successfully curbed the chronic pain epidemic.

These failures are all the more striking since, to the individual experiencing pain, no experience seems more obvious, immediate, and given. Indeed, pain seems like an instance *par excellence* of immaculate perception, i.e., an experience so clear and distinct that the person cannot fail to know what it is like. Nevertheless, from a clinician's point of view, pain seems to be one of the most complicated conditions to recognize and treat. Healthcare providers struggle to formulate reliable tools to assess pain and respond to it in a way that actually helps patients (6). This situation is intuitively odd: If pain is so certain and real, why do we constantly fail at dealing with it? The fact that matching the right patient with the right therapy is so challenging, despite an arsenal of pharmacological, psychological, surgical, neuromodulatory, and many other available forms of management, speaks to this lacuna. Worse yet, a grand unified theory of pain that explains all types of pain, harmonizes verbal pain reports and behaviors, and connects physiological processes with experiential states is sorely lacking.

All of this is to say, *we do not know how to deal with pain, nor do we have an apt theoretical account of it*. Such empirical realities and theoretical gaps lend support to our overall contention that something has gone badly wrong at a fundamental level. In the following Sections 1.2–1.6, we explore possible causes of this error—that we are all trapped by the long shadow of Descartes and forced to think of pain as either a ghost of the mind or an error in the machine. As we shall see, it is only by rejecting this framework that we can hope to make progress to better deal with pain.

### 1.2 Origins and theses of pain privacy

The terms “private,” “subjective,” or “personal” are often used interchangeably, raising some problematic equivocations we foreground throughout this paper. In any case, let us focus for now on the claim that pain is private and explore its ramifications. Specifically, we will discuss the origins of this claim, articulate how it functions, and consider how it continues to influence clinical practice and research. Finally, we will examine the ground truth of pain for such a privacy account.

To begin, the idea that pain is inherently private is pervasive, although rather counterintuitive. People “in the wild” do not go around insisting that I cannot have *their* (private) pain the way they might insist that I cannot have *their* (private) car. Indeed, they seem to want to share their pain desperately, as anyone who has worked in a hospital can readily attest to. Nevertheless, some of the philosophical and medical literature seems inexorably committed to this thesis [e.g., (7–9)]. This raises the following question: Where did pain's assumed privacy come from?

It seems to us that the privacy of pain entered into both the clinical and research literature decades ago. On the research side, a crucial source was the pathbreaking work of Henry Beecher, the first person we know of to begin using self-reported pain scores as a crucial independent measurement of pain. Beecher contended that a key difficulty researchers face is that it seems “paradoxical to speak… of measuring something which cannot be satisfactorily defined– and if this were true it would be a paradox or nonsense or both. The fact is, pain is defined introspectively by every man. The difficulty comes in verbalizing this well known experience… in saying *what* it is” (1). On the clinical side, perhaps the most consequential definition of pain stems from Margo McCaffery's claim that pain is “whatever the experiencing person says it is, existing whenever and wherever the person says it does” (2) and her subsequent advocacy for self-reported pain intensity scores as the basis for pain management. Notice that these proposals find a striking harmony with one another. Both insist that pain is private *and* measurable, both assume that a patient cannot be wrong about her self-reported pain, and both set the self-reported score as the “gold standard” for understanding and treating pain. Let us call such a view, the privacy view.

Crucially, the privacy view is underdeveloped, as it attempts to align semantic considerations of “pain,” epistemic issues about accessing and assessing pain, and ontological considerations about the nature of pain into an interdependent and mutually reinforcing framework. We will examine each in turn and their interactions and eventually show what the privacy view really amounts to.

At the semantic level, the basic thought is that pain-talk describes a person's experience of pain, including the unpleasantness of the sensory experience, along with the ache and the suffering. In turn, this construal implies that “pain” refers “to what only the speaker can know – to his immediate private sensations. So another person cannot understand the language [as she cannot grasp the concepts involved]” (10). This seems to capture what both Beecher and McCaffery were after. Effectively, the meaning of “pain” stems from its referent. And since its referent is an experience, it follows that what “pain” means depends on which experience it is clipped to.

In turn, the claim that the semantics of “pain” turn on experience readily integrates with epistemic considerations of how we access and assess the experience. It seems clear that the experience of pain is inherently first-person singular. Effectively, only *I* can have *my* pain. In turn, this readily suggests an epistemic thesis. To wit, since only I have my pain, only I know what my pain feels like. Effectively, I have a pain, I am aware of this pain, and it is in virtue of my awareness of this pain that I know *what* it is like. Indeed, this is often pressed even further. Specifically, many philosophers claim that the experience, in and of itself, generates the concepts I use to understand it [e.g., (8, 9, 11)]. Now, how this generation works and what it implies is hotly debated (ibid). But most agree that my concept of pain is true of my experience because it is my experience that gives rise to the concept in the first place. In turn, this easily integrates with the semantic view mentioned above. “Pain” is true of certain experiences for me because my experiences generate the concept that is true of it.

Finally, these two claims entwine with a radical ontological proposal. This proposal claims that, for pain, esse est percipi (to be is to be perceived). In other words, what makes a given experience painful is the fact that *I find it so*. In this, it is my “inner” assessment that determines what pain is. Notice that this takes a rather trivial remark about pain's ontology and radicalizes it. To see this, start with the truism that pain (and other such experiential states) is ontologically subjective in that it depends on consciousness. Bluntly, no consciousness entails no pain experience [cf. e.g., (12, 13)]. This is a perfectly clear and obviously correct use of “subjective.” However, in itself, this does not make pain (or other such experiences) turn on self-assessment. For example, major depressive disorder is certainly ontologically subjective in that no people imply no depressed people. Nevertheless, the *Diagnostic and Statistical Manual* revised fifth edition takes pains (pun intended) to provide criteria, like weight loss or sleep disturbances, that do not simply depend on the person's self-assessment of her emotional experiences [cf. (12, 14)]. Indeed, a trained psychiatrist can correct a patient's self-diagnosis, regardless of how honestly she believes that she is depressed. In this, though the patient may have the first word, she does not have the last one. However, from the privacy view, the subjective dependency is radicalized into subjective self-determination. It is the patient, and *only* her, who can determine if she has pain. In other words, it is her perception of her experience that makes the experience what it is. Or, to come at the same point in a different way, the fact that I feel pain is existentially committing in that it implies I have pain. As it were, my feeling (and nothing else) determines reality, a radical departure from other subjective conditions like depression or anorexia nervosa.

In turn, this radical ontological claim gains support from and underwrites the semantic and epistemic claims mentioned above. Precisely, since my consciousness itself determines what pain is, it follows that only my consciousness can know what counts as pain. And it is this knowledge that then provides criteria for what “pain” is true of. Or, working in the reverse, since what “pain” is true of is ultimately an experience, and since only I know my experiences because I alone can have them, it follows that my perception of experience is a precondition for its existence. **In either direction, we see that the privacy view integrates the semantic, epistemic, and ontological level into a (seemingly) coherent and cogent framework for reflecting on pain**.

And it seems to us that the privacy view is often tacitly or explicitly assumed in many accounts of pain in both the clinical and research contexts. From the research perspective, supposedly self-reported pain score intensity change is “likely… the most commonly pre-specified primary outcome in chronic pain trials” (15). Indeed, almost every study on pain takes it for granted that (i) a subject *can* measure a subjective experience (ii) that this measurement (at least for some scales like the VAS) is cardinal and (iii) that the measurement is *certain*. From a clinical perspective, the dictate that a patient's self-reported pain score is equivalent to the “gold standard” of pain assessment is still a dogma in nursing, as evidenced by the stress given to it in the *Nursing Diagnosis Handbook*, a text all student nurses in the USA are trained in (15). In both cases, the privacy of pain connects with the idea that a patient has the final say. Effectively, a patient says a numeral, and it is this, and only this, that effectively anchors decisions in research and clinical contexts. Finally, given this, the privacy view defines the ground truth of pain assessment in a very particular way, i.e., irrespective of context, inter- and intrapersonal factors, and (at the absolute best!) only weakly correlates with physiological features.

### 1.3 Ghosts cannot hurt: challenge for the privacy view

Given the above, it is clear that this privacy view sets up a very particular way of conceiving of pain. Briefly, some experience counts as pain if and only if the person feels it does. In itself, this does not yet show that this account is fatally flawed. Indeed, maybe each person has her own private language, and we can somehow come to cotton on to parts of this private language via halting analogies (or whatever). However, it is here that Wittgenstein's polyphonic assault on the very idea of a private language shows us why the privacy view is incoherent and shatters pain assessment itself.

However, before we begin, a note on the possible interpretations of Wittgenstein's private language discussion (PLD) is in order so that we can locate ourselves in the philosophical literature. There are roughly four major interpretations of the PLD in the offing at the moment. One claims that the entire discussion is simply flawed. Often, this interpretation claims that PLD presupposes some now-defunct philosophical thesis (e.g., a verificationist theory of meaning) and uses this to claim that the PLD is simply wrong [e.g., (8, 9)]. We reject this interpretation as both unhelpful and rather uncharitable. A second interpretation argues that PLD is apiece with Wittgenstein's broader skeptical assault on the very idea that meaning has anything to do with facts and his turn to communal endorsement as bedrock [e.g., (16, 17)]. It seems to us that this account appreciates the depth of the rule-following objection and sees, pace the first interpretation, how truly radical PLD is. Nevertheless, the skeptical solution it offers, i.e., that “pain” applies to whatever my community says it does, seems to trivialize PLD. A third interpretation casts PLD as a sort of conceptual cartography that has two interdependent aims. Positively, Wittgenstein aims to map out the constitutive interdependent use-rules for “pain,” thereby disclosing the bounds of sense in this particular domain. Negatively, Wittgenstein wishes to show that attempts to use “pain” referring to rarified experiential ghosts (or reified neurological machines) violate these rules and so inevitably generate non-sense [e.g. (18)]. Although we are sympathetic to some aspects of this interpretation and draw from some of its positive account, we reject it as overly prescriptive and inflexible. Indeed, we think a key lesson to learn about the bounds of sense is that it can and does change—Euclidian geometry *was the* geometry until it wasn’t, a point that caused some problems for Kant's similar attempts to crystalize the limits of intuition. A final interpretation views PLD as a form of careful philosophical anthropology wherein the meaning of “pain” and the nature of the experience it links to requires careful attention to the roles it plays in human forms of life [e.g., (19–21)]. Such an interpretation emphasizes that it is *persons* who have pains and so understanding pain requires getting clear on why and how people have it. It is this interpretation that we most closely follow.

With this (brief!) literature review in view, let us turn to the actual argument. To begin, to argue that pain is inherently private is to accept that pain expressions are accidental vis-à-vis the essential experience. This is simply because what determines if some x counts as a pain is the essence of the experience itself, not its expressions. Again, this is in marked contrast to major depressive disorder wherein we expect and exploit indicators to guide diagnosis. In any case, to show the problems with the privacy view, we break it into two related, misguided claims. (a) First, there purely private pain is only accidentally related to expression in behaviors, pain-talk, etc. (b) Second, there is no public behavior, pain-talk, etc., that can convey the private experience of pain. Let us discuss and refute each in turn, while exploring the intractable difficulties they cause in a clinical context (and beyond). Note that we substantiate many of these brief remarks in the next section, by pairing them with cutting-edge neuroscientific research.

(a) The claim that private pain is only accidentally related to behaviors, pain-talk, etc., can be unpacked conceptually, logically, and normatively.

Conceptually, by assumption, private pain in and of itself provides me with all the resources I need to recognize it. Effectively, how I react to noxious stimuli is irrelevant to categorizing some experience as an instance of pain. This is because an *essential* property of the pain experience is that it *feels painful*, not described in behavioral or C-fiber terms (22). Therefore, I ought to know that an experience is pain *simply* in virtue of the experience itself, *not* because of how the world is, how my body reacts, my past experiences, my expectations, my socialization, how others interact with me, etc. Instead, I know that an experience is painful based simply and solely on the brute *givenness* of the experience itself, and I’m intrinsically equipped with everything that is needed for me to make this determination. To put it crudely, pain makes sense of itself to me intrinsically and with absolute certainty. A bit more formally, the experience of pain has an essential property of *feeling like this*, and it is solely in terms of this property that I classify some experience as pain. In turn, this implies that everything else, e.g., stimuli, verbalization, brain activity, etc., is divided out as irrelevant for recognizing pain.

This position faces serious challenges. Empirically, what pain feels like is in fact highly contextual and strongly dependent on “outer” factors, as systematic reviews of the neuroscience literature suggest (23). This naturally suggests that the experience of pain is not totally “self-contained” and “inner.” For example, and consistent with the neuroscientific findings, one qualitative study found that women in labor who feel socially supported experience their pains in a radically different way than those who do not (24)]. Crucially, what makes the difference here is not the brute occurrent sensation but what the person takes the sensation to be *about*, i.e., how they interpret it. And this interpretation turns on their overall context and this informs how they understand their pain. And this may generalize. To give another example, it seems extremely likely that chest pain in a person with long-standing and well-known stable angina is experienced very differently from a person for whom chest pain is totally new and utterly terrifying. And, again, what might make this difference are contextually varied “outer” beliefs about what the chest pain *indicates* [cf. (25)]. To presage an idea we develop more fully in Section 1.8, part of what occurs in these cases is that different people place different “bets,” based on different context-sensitive priors, and these “bets” change how they experience the pain. In any case, “outer” context matters for “inner” experiences because we understand brute physiological changes in terms of *what* they respond to, not just their “givenness.” Chest pain as a sign of mild ischemia is very different from chest pain as a harbinger of the Grim Reaper coming to collect his due; birth pain as a productive part of having a child with a loving partner is different from birth pain in isolation from such support, etc. The “inner” depends on the “outer” because we leverage the latter to make sense of the former – the sensation is “always already” about something, a response to something, and this contextual something helps shape the experience.

Next, the links between experience and concepts are fraught. Recall that the basic idea is that an experience engenders the concepts that I use to understand the experience itself. This *might* work for simple, uniform, and unidimensional experiences like seeing a specific shade of blue. The problem is that the experience of pain is complex, dynamic, and multidimensional. Indeed, the same experience seems to give rise to concepts of intensity, duration, quality, etc. And this leads to a simple question. Are these concepts the same or different? On the one hand, it seems like they must be the same as, *prima facie*, they are generated in the same way by the same experience. On the other hand, they must be different as the same concept of intensity can be applied to an aching pain and a burning pain occurring simultaneously in different parts of the body.

Finally, this difficulty seems to spring from the ontology of pain itself. Specifically, is our experience of pain essentially or accidentally multidimensional? **If accidental, then why do we not experience pure pain intensities or pure pain qualities alone? If essential, then what unifies the medley and brings about “suffering”?** If there is an external property that unifies the medley, we face a version of Bradley's regress, i.e., a class of regress arguments that suggests that unity cannot just be another property. For us, the regress goes as follows. Assume that some pain is a collection of properties like intensity, location, duration, etc., that are held together in our experience of pain. This raises the question of what holds these properties together. And one might answer that there is an external property, i.e., a property that is not itself intensity, duration, etc., that unifies the properties together in the right way. As it were, the external property is akin to the glue that connects properties like intensity, quality, etc., together into our experience of pain. However, this sets up a regress as now it seems like we need an additional external property to unify the original external property to the other properties so that they can be unified, etc. As it were, we need glue to glue the glue, ad infinitum. Alternatively, one might think that the multidimensional experience of pain is inherently unified. However, it then becomes unclear how each aspect of the pain experience can vary independently of other aspects. Indeed, *prima facie*, the *same* (?) headache can be severe at one moment and be(come?) mild the next. Add to both the fact that I am working at a strange third-order level (e.g., properties of properties of experiences, aspects of properties of experiences, etc.) and one rightly may become nervous that identity conditions themselves come under strain as it is not entirely clear what would make an aspect of a property identical to another aspect. A third move is to say that we are thinking about the multidimensionality of the pain experience in the wrong way. This suggests that the unity may be neither another property nor an intrinsic part of the experience but stems from something else. And, plausibly, whatever this “something else” is, it is not something that is on all-fours with the brute “givenness” of experience. And this suggests, again, the need for the “outer.”

Logically, it is clear that public expression is neither necessary nor sufficient for private pain. It is not necessary because people have private pain without public expression. To take a well-worn example from philosophy, one can imagine super-duper-Spartans who experience private pain but have been socialized in such a way that they never express it in words or behaviors (26). More intuitively, most people have experienced private pain and yet resisted expressing it for whatever reason. It is insufficient because a well-trained actor can perfectly mimic all the public behaviors of pain (and win an Oscar for convincingly communicating that pain experience), yet lack the experience.

Although this point stands in itself, one worry is that it can be (sometimes is, in philosophy) pressed too far. Nothing in deductive logic entails that a high fever is a sign of the flu. But moving from this point to the further contention that high fevers have nothing to do with the flu is a gross misuse of deductive logic. Not all relations are logical entailments [cf. (27)]. Indeed, to presage a bit, Bayesian probability is perfectly formal and yet not deductive *per se*.

Normatively, one can argue that the presence of private pain does not, in and of itself, imply any further actions I *should* take. This may seem odd. But consider Hume's well-known dictum that one cannot derive an “ought” from an “is.” The underlying thought is that “is” statements merely describe the world as being thus-and-so, and the world being thus-and-so, in itself, has no normative implications. Now, since the privacy of pain stems in part from the view that pain-talk is descriptive, it follows that my pain-talk merely informs you of a fact about me, akin to my height or my genetic code. Moreover, arguably, my pain experience is merely my mind/brain/sub-personal processor gathering information so as to represent my bodily state to me. Again, this suggests that pain, in itself, has no normative consequences. Granting this, it follows that talking, crying, screaming, etc., in response to pain are *not* unmediated consequences of the pain experience.

However, tellingly, pain *is* consequential, urgent to a high degree, and deeply connected to expressions and actions. *Pain highjacks the human brain, like a loud alarm bell that won’t shut off until a remedial action is taken*. Therefore, it is almost impossible to uncouple the pain experience from the *doing about it.* Indeed, again presaging a bit, if we adopt a broadly Bayesian perspective, beliefs about pain, expressions of it, etc. are best construed as action-guiding “bets,” and so they are already shot through with normativity. If I strongly believe it will snow tomorrow, then I ought to be willing to put some money on it (as well as park my car in the garage). **And if I tell you about my pain, then I am betting you can help me with it**. It also suggests that pain experiences are not the brain/mind passively registering information about the bodily world. Instead, it may be that these normative response patterns account for the medley of experiences people classify as pain. In other words, the extension of what people count as pain is *not* determined by some essential property that all and only certain types of experience have but also depends on a normative evaluation of *how* the experience inter-depends with the patterned engagements manifested in response to it. If you like, “pain” is a thick concept [e.g., (28–30)]. Or, more intuitively, I make sense of an experience not only via an essential property it has, but also based on how it inter-depends with my world. A rapid heart rate may be experienced as lust or fear, depending on the presence or absence of a nubile confederate or a beasty, an old point from social psychology and classical phenomenology [e.g., (31, 32)]. And, similarly, being whipped may be experienced as pain, pleasure, or *Lacanian jouissance* if one is in a BDSM club. Note that, for social beings, this all makes good evolutionary sense. The experience compels me to engage in outward-projecting behavioral traits that are beneficial to the community and engrained within distinct neural circuits in the brain (see below). These points elaborate on the absurdity of private pain that requires no public expression, whereas my pain does have a real and measurable impact on the world.

(b) The claim that pain-talk, behaviors, etc. cannot convey pain is a simple consequence of privacy.
  Succinctly, since it is assumed that pain-talk, behaviors, etc. do not transmit your experience of pain to me, it follows that they can give me no insight into your experience of pain. More carefully, one is often told that one infers the presence of pain in another from evidence, including specific behaviors, reports patients make, etc. There is a core flaw with such an account. Specifically, to infer from A to B requires a prior grasp of the propositions involved. So, what is the proposition that I am to infer when watching the child weep? Obviously, by privacy, I cannot infer that she has *my* pain as she *cannot* have my pain. So, I am supposed to infer that she has an experience that is similar to or like mine. However, to say that X is similar to Y requires that I access X and Y independently and then discover some shared properties. I cannot access the child's experience. And an essential property of my pain is that it is *mine*. So, neither the object nor the property works right. Hence, this collapses.

This last point, in particular, is devastating. **The privacy of pain leads to the conclusion that I can never access, assess, understand, or treat another's X** (pain? A “similar” experience? No experience at all?). In turn, this makes literal non-sense of pain-talk as I have no idea what someone is describing when they say, “I have a pain.” Indeed, one is forced to ask “[w]hat gives us *so much as the idea* that beings, things, can feel [pain]?” [(33)]. It is no wonder healthcare has failed so badly at managing pain!

All these points raise grave concerns for the privacy's views claim that the ground truth of pain is the feeling of it. Indeed, despite the fact that Beecher was forced into something like this position, he was skeptical of the idea that we all “just know” what pain is by experiencing it. All of this is to say, pace Beecher's gambit, it is paradoxical or nonsensical to claim that we can measure something we cannot define.

In closing, it is important to notice that dissatisfaction with this account of the ground truth of pain is becoming more and more widespread in both clinical and research contexts. And although our discussion may be slightly more philosophical than many criticisms, it has become increasingly clear to most healthcare practitioners that allowing patients to dictate their treatment in light of their feelings leads to a host of problems. It is to a countervailing proposal we now turn, one that, as Newton would have it, is the equal but opposite dialectical reaction to privacy.

### 1.4 Cartesian machines in pain

If the above section is persuasive, it is clear that the ground truth of pain cannot be private. And this raises anew the question of what it should be. **Recently, some researchers have proposed that we should link the ground truth of pain *not* to private experiences, but to measurable biomarkers**. In this, these researchers propose a *reductive* use of biomarkers in that they are the key to objective pain assessment. Given this reductive use, they recast the ground truth of pain in objective terms. Specifically, they claim that a patient is in pain if and only if certain biomarkers are present to some sufficient degree. Let us discuss why this view is gaining increasing traction. Then, let us examine the holy grail of this account, the quest for a “pain meter.” This sets the stage for the next section.

The reductive use of biomarkers to explicate the ground truth of pain connects with a broader shift in healthcare generally. To wit, healthcare in the U.S. is progressively shifting to a value-based care model that focuses on quality, provider performance, and patient experience. Given this, the abysmal failure of the privacy view of pain and the non-reliable diagnostic procedures it relies on are simply not tenable. Indeed, as the number of patients seeking pain relief rises, personalized therapies for chronic pain are lacking, readmission and opioid misuse are increasing, thereby lowering patient satisfaction and contributing to healthcare disparities. As profit margins shrink, burned-out providers have less time to spend on in-depth review of the medical history and electronic records. In turn, patients increasingly feel like their testimony is undervalued and their perspective discounted by busy providers who do not listen to them. And all of this triggers a vicious cycle wherein providers discount patient testimony and patients realize that providers no longer pay attention to their needs, wants, and values. The result is that value-based care is undermined by the healthcare system's inability to manage pain.

**Figure 1:**
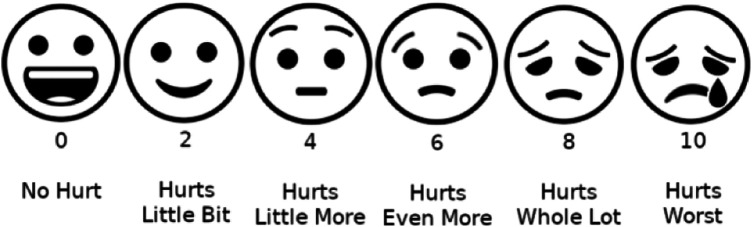
Wong-Baker scale for self-reporting of pain.

**Given this, it is clear that locating and correcting the root cause of this failure is of paramount importance. And the reductive view claims to locate the source of these problems in the individual's exclusive “ownership” or privacy of the pain experience**. In this, the reductive view is an outright rejection of the privacy view and the Cartesian ghosts that haunt it. Moreover, the reductive view explains the failures mentioned in both Section 1.1 and 1.3 as simple consequences of bad diagnostic techniques. For example, the privacy view tends to forcibly reduce the complex and multidimensional experience of pain to the crudely simplistic visual analog scale (VAS) or the overtly childish “smiley and frowning” Wong–Baker scale (see Figure 1), which fixates solely on intensity. Or else it starts ramming its head into a wall trying to make sense of aspects of properties of experience. And this is exactly because the privacy view leads one to conclude that the experience, and nothing else, determines if a person has pain. Such a view rapidly degenerates into a fatuous “medicine by emojis.”

From here, the reductive use seeks to overcome “medicine by emojis” by a wholesale replacement of privacy with objectivity. To do so, it reconceptualizes pain's ground truth in terms of *measurable* objective indicators, whether or not the patient is aware of (or even understands) them. To give a concrete example, consider a reductive use of an opioid compound that binds to the family of *μ* opioid receptors. Given this, we can measure the effects of this compound in terms of receptor occupancy. And, since opioids are known to reduce pain, it is logical to assume that the physiological state of effective receptor occupancy will likely translate to a mental state of reduced pain. Conversely, it also stands to reason that more unbound μ opioid receptors will lead to more pain. Simple, clear, and objectively measurable in either case.

In turn, this reductive use of biomarkers has given rise to a quest for “pain meters” that resemble blood tests for diabetes or thermometers for fever. The hope is that such “pain meters” will allow for more reliable diagnoses and effective treatments, thereby reducing patient suffering, provider stress, and more robust “bottom lines.” To see how such “pain meters” might work, consider an analogy. A provider does *not* diagnose hyperglycemia by signs like frequent urination, or odd testimony from the patient that he has “300 too many sugars in my blood.” And she does *not* treat it by arbitrarily selecting 5 units of insulin, giving it to the patient, and hoping this will fix the problem. Instead, she uses a glucometer that provides an objective measurement of the serum glucose level coupled with evidence-based nomograms that precisely specify the amount of insulin needed to reduce it to an acceptable range. Notice that the only relevant information the provider needs or should consider is the glucometer's reading, not symptoms, signs, patient testimony, etc. Indeed, a patient may well be hyperglycemic without any symptoms at all. Such an approach ameliorates provider stress by removing the guesswork, helps the patient by targeting the underlying cause of her condition and fosters value-based care by providing rapid and reliable treatment.

Similarly, it is claimed that a “pain meter” will bypass the bottleneck of pain privacy and enable a provider to identify the pathophysiological type and amount of pain a patient has with certainty and proffer evidence-based nomograms tailor-made to the specific pain condition. Indeed, such an objective approach promises to be simple, reliable, and by the numbers. Moreover, it can easily be incorporated into the evidence-based framework of medicine [cf. (34)]. And, finally, it holds out the promise of removing the messy business of actually listening to the patient's reports of symptoms or careful observations of potential signs of pain. Hence, what matters is the mechanics of the machine, not the ghosts of the mind.

In sum, frustrated researchers might be lured into developing biomarkers as a key to more reliable, objective pain assessment. In itself, we wholly endorse and welcome this turn, cautiously. However, some researchers push further, recasting the ground truth of pain in terms of biomarkers and claiming that a “pain meter” is the magic solution to all our problems. In this, they claim that someone is in pain if and only if some set of biomarkers are found in a sufficient quantity or magnitude. It is this claim we find misguided. To see why, let us turn to the next section.

### 1.5 Machines cannot feel: problems with the reductive use of biomarkers

**Again, though we welcome research into biomarkers and happily hope that they will give us far more reliable pain diagnostics than, e.g., smiley faces, we think that the attempt to reduce the ground truth of pain *solely* to biomarkers is about as absurd as the quest to discover the west pole!** There are several reasons for this.

First, there is the fact that the reductive use of biomarkers implies that we can diagnose pain without any reference to experience at all. However, this seems to us to be a category mistake. To see this, imagine that researchers have identified a reliable set of histological -omic biomarkers for a certain type of pain. If these biomarkers are all that is required to diagnose pain, it seems to follow that a test tube filled with them is in pain. Obviously, this is absurd. However, it seems to us that the same basic mistake occurs at seemingly more sophisticated levels as well. For example, some claim that if certain neurological pathways are activated, then there is pain. However, one can imagine an *in silico* replica of these pathways causing the computer pain, an absurd claim.

Second, relatedly, such a reductive proposal both flies in the face of cutting-edge neuroscience of pain and issues strange promissory checks that no one knows how to cash. To begin, detecting changes in the environment is the crucial first step in directing our attention and entry of select stimuli to awareness. Of all sensory stimuli, accurate and reliable detection of noxious stimuli capable of producing pain is arguably the most immediately and directly consequential to wellbeing. Pain depends critically on the accurate and rapid detection of noxious stimuli. The first step in this process is mediated by neural activity conveying sensory signals through peripheral and spinal nociceptive pathways (35). Perception of the features and severity of pain involves additional processing by distributed neural networks in the brain that produce a multidimensional pain experience. Magnetic resonance imaging (MRI) and other electrophysiological studies over the past decade have postulated that a “pain matrix” comprised of cortical and subcortical structures is activated during pain (36, 37). Within this matrix, the primary somatosensory cortex (S1) has been shown to contribute to the early part of the cortical response elicited by noxious stimuli (38), mediating the initial localization and discrimination of these stimuli (39, 40). Hence, the representation of nociceptive stimuli in the neocortex is only the *initial* step in the pain experience.

However, pain is not simply a deterministic (i.e., objective) representation or an image replica of a noxious event in the environment, as suggested by the reductive use of biomarkers. In fact, the representation of nociceptive stimuli in neural circuits of the mammalian brain is not fully understood, and the concept of a “pain matrix” has been debated and refuted (36, 37). Detection is only the first step in the multidimensional pain experience. That said, current scientific knowledge in this field fails to explain how neural activity in the brain is “magically” transformed into emergent properties underlying the conscious experience of pain. Overall, studies rarely account for external and internal variables known to significantly impact our interpretation of a nociceptive stimulus as painful. In turn, this current inability to deal with the relevant variables properly often leads researchers back to the Cartesian view of pain, i.e., as a feedforward system that copies nociceptive activity and pastes it into an immediate pain perception that I then refer to.

The result of this is a strange amalgamation of two claims. First, we are issued a promissory note that, somehow, future neuroscience will explain the alchemic transmutation of brain processes into experiences. Second, since no one has any idea of how science can explain alchemy, researchers are often forced to admit that at present (and for the foreseeable future), we must simply accept that we have no bridge between nociceptive activity and the feedforward system and conscious experience. Ironically, here a dialectical reversal occurs where the reductive biomarker view may well be forced back into the privacy view it so adamantly opposed.

Third, the reductive use of biomarkers is actually ill at ease with value-centered care, despite claims to the contrary. Specifically, and especially if one insists on delivering value-based care, then dismissing outright what a patient says about their current experience is simply not tenable. Indeed, ignoring what a patient says about themselves because, e.g., the fMRI failed to show that the relevant pathways were active will not lead to better care. Moreover, and again with some irony, it seems to us that the reductive use of biomarkers repeats the same mistakes of the privacy view in a different form. Attempts to reduce a multidimensional experience to a smiley face are absurd. However, by parity of reason, attempts to reduce it to a numeral that some “pain meter” displays is equally mad.

Fourth, pursuant to this, the reductive use of biomarkers (and the privacy view of pain) exculpates clinicians and researchers from the difficult task of actually *listening* to the person in question or being liable to misinterpretation of the person's report. A clinician does not have to take very seriously a patient's reports concerning how annoying frequent urination is during hyperglycemia. They just need to check glucose and give insulin. By analogy, the same should be true for “pain meters.” However, to hint at a track we will develop more later, we do not think that dismissing a patient's pain narrative is viable.

Finally, conceptually, consider one implication of setting the reductive use of biomarkers as the clinical “gold standard.” To begin, a person is hyperglycemic if and only if her serum glucose level is above, e.g., 125 mg/dl (per Cleveland Clinic standards) regardless of any other signs, symptoms, reports, etc. By analogy, a person has pain if and only if the “pain meter” detects some empirical indicators beyond a certain normal range regardless of any signs, symptoms, reports, etc. Such a view goes badly wrong in both directions. On the one hand, it implies that someone can have pain without experiencing it. Even granting that this is conceptually coherent (we do not think it is), a problematic consequence is that she may be given a drug, based on a nomogram, to reduce a pain she does not feel. On the other hand, it implies that someone without the relevant objective indicators is not in pain, regardless of how they feel. Such a contention threatens to damage an already fraught situation as a provider may outright reject a patient's testimony because it does not align with the reading from the “pain meter.” Needless to say, this neither facilitates a good patient-provider relationship nor less does it further the goals of value-based care.

That said, we think that **the project of identifying biomarkers is laudable, *provided that the quests to privatize or objectify pain are dropped***. We return to this in a moment. However, for now, let us summarize the negative portion of this paper.

### 1.6 Descartes's long shadow: ghosts, machines, and “pictures” of language

The above sections discussed two different accounts of pain, the privacy view, and the reductive use of biomarkers. And it argued that both face deep problems. Moreover, the last section hinted at something important. To wit, the reductive use of biomarkers seems to repeat in a different key the same mistakes as the privacy view. This seems surprising. However, we believe it is due to the fact that pain researchers and clinicians are stuck between the horns of a (false!) antinomy. Specifically, researchers and theorists are forced to choose between ghosts or machines, privacy or objectivity, feelings or biomarkers. And when one choice fails, they cycle back to the opposite (and equally untenable) extreme. Thus, psychophysics promised to make sense of pain, but privacy rejected this, only to be rejected in turn by more sophisticated objectifying accounts that generate their own issues, giving rise to more sophisticated versions of privacy that fail, and so on. Here, strangely, equally untenable extremes feed into each other.

To escape this, it is important to clarify *why* we are trapped in this false dichotomy between experiential spooks and physiological systems. And we think that Wittgenstein (again) provides a powerful explication of why we are caught in this fly bottle and how to find our way out of it.

According to Wittgenstein, our seemingly intractable problems stem from a particular “picture” of how pain-talk works. According to Wittgenstein, the default “picture” of language that we often begin with is that the primary function of language is to *describe* some facet of the world as being thus-and-so. In turn, this suggests that when someone says, “I have a pain,” they merely *describe* how things stand with them like “I have a pin” describes an item they currently possess. In turn, this connects with Descartes's construal of mental talk generally as describing “inner” properties, states, objects, etc., of the “thinking stuff” [e.g., (33)]. Notice that this “picture” suggests that the role of the mind/brain is passively registering some already given facet of reality rather than, e.g., actively contributing to it (as discussed below). As innocuous as it sounds, making this “picture” of pain-talk work requires several commitments, forcing one to wrongly conclude that pain is either a ghost or a machine.

According to this “picture,” pain-talk must describe some independent facet of the world. However, here, a problem emerges, as it seems like trying to peg “I have a pain” to tissue damage is flawed. At the extreme end, people with phantom pain lack tissue that can be damaged, yet still insist that they have pain in the lacking tissue. In a less exotic key, headaches, neuropathic pain, complex regional pain syndrome, etc., are all independent of actual bumps and bruises. It also seems like crude forms of behaviorism are wrong too. “Pain does not describe behaviors if, for nothing else, it seems like people apply it without reference to behaviors.” From here, since “I have a pain” needs to be describing something, and since there is no obvious something to point at, the “picture” demands that we look in more exotic places. It is exactly here that we seem forced to choose between ghosts or machines. For ghosts, the basic thought is that “pain” refers to an experience. And this sets in motion the privacy view articulated in Section 1.2 and leads to the difficulties in Section 1.3. For machines, the reductive use of biomarkers renders “pain” some objective facet or other. This kicks off the dream of a “pain meter” discussed in Section 1.4, turning into a nightmare in Section 1.5. In both cases, what drives both the privacy view and the reductive use of biomarkers forward is the presupposition that “pain” refers to some static, context invariant, and pregiven “thing.” And the deep mistake we make is to assume that when language suggests a body and we find none, we must invent it.

And Wittgenstein's proposed way out here is at once straightforward and radical. He suggests that pain-talk does not describe and that our pain experience is not simply passively registering via introspection how things stand with us. Indeed, **Wittgenstein begins to show that pain and our talk about it are dynamic and “constructed,” not static and “given.”** And it is this insight that sets the stage for our positive proposal standing on philosophical and neuroscientific grounds.

### 1.7 Definition of pain biomarkers according to FDA guidelines

Let us begin our positive proposal with a discussion of what biomarkers actually are and what we can reasonably expected from them. Several branches of government are promoting research that improves our understanding of the basic mechanisms of pain, and encouraging new conceptual frameworks that could be leveraged towards the development of reliable metrics for diagnostics that guide effective therapies. Major funding initiatives at the National Institutes of Health (NIH) advocate for research that captures the holistic pain experience, taking into consideration individual differences. There has also been emphasis on the importance and necessity of acknowledging the subjective elements of that experience, and the use of objective measures as *complementary* rather than exclusive metrics. These measures are collectively referred to as “biomarkers.” Indeed, Saab and colleagues noted the “*importance of [pain] biomarkers that complement the patient's self-report*,” further explaining that pain assessment is the process of approximating a person's self-narrative or “ground truth” (41). Hence, **pain biomarkers must be grounded in the patient's narrative and behavior towards others** when relaying the experience of pain. Indeed, this account fundamentally alters the ground truth of pain. It rejects the hope that we can find simple, reductive, biconditionals of the form x is pain if and only if y. Instead, it emphasizes that pain is best understood in terms of its roles in human forms of life, roles that a patient's narrative foregrounds and roles that biomarkers can help to clarify. Note also that this definition of biomarkers allows us to expand the scope of the pain assessment metrics while avoiding the problematic thought of “cutting the patient out of the loop.”

A few use cases of biomarkers are worth noting. According to the FDA's Biomarkers, EndpointS, and other Tools (BEST) guidelines, pain biomarkers that fall in the category of target engagement indicate that a drug has reached its physiological target in the body and, in doing so, resulted in a measurable effect. To clarify how the supplementary use of biomarkers is different than the reductive use, let us return to the example of an opioid compound that binds to the family of μ opioid receptors. As noted, such opioids allow for a measurable effect here defined as receptor occupancy. Crucially, even though the target can be shown to be effectively engaged, the ultimate goal of reducing pain can only be confirmed by the patient's testimonial, which is influenced (again!) by multiple psychological, emotional, and contextual factors. Therefore, target engagement biomarkers should *not* be confused with objective pain diagnostics. Similarly, pain biomarkers that fall in the categories of predicting response to therapy, subject selection, and safety monitoring do not measure an individual's pain experience *per se* (i.e., they are not designed to diagnose pain). In fact, **there is no valid category or class of biomarkers that can reliably measure the subjective experience of pain***.* This furthers our point that the quest for a “pain meter” is a non-starter as the ground truth always inexorably depends on a patient's experience and her testimony concerning it. In the final analysis, it is always the patient's narrative, not the presence of biomarkers, that determines how her pain is and which treatment alleviates it.

Hence, biomarkers indeed hold great promise for helping us overcome “the science by emojis” account that is so common. However, they can only play this role within the context of the patient's narrative and, relatedly, her overall form of life. Indeed, this partly explains why the claim that a test tube filled with -ominic biomarkers is not in pain *per se*. What matters here, what makes these biomarkers indicators of pain, is their contextual role in the life of the person, not simply their biochemistry. And the same is true, mutatis mutandis, for neuroscience. It is to this point we now turn.

### 1.8 Pain experiences are subjective *because* they are actively shaped by the brain

We know that for a given noxious stimulus, pain is modulated by events in the environment that compete for our attention and internal biological variables such as inflammation, or prior *brain state*. What do we mean by *brain state*?

The brain of an awake and attentive person is not passively awaiting external stimuli to rescue it from idleness. Even while at rest, the human brain exhibits constantly changing patterns of cortical activity across distributed networks. The meaning of this seemingly random or stochastic brain activity remains a matter of debate. William James described this phenomenon as a natural state of unstable equilibrium that is best adaptable to the minutest alterations in the environing circumstances (42). In the context of this paper, we define *brain state* as the spontaneous activity in the brain while the subject is not actively engaged in behavioral or mental tasks. For example, one of the brain state networks, also known as the default-mode network, is thought to reflect a person's state of mind wandering, i.e., “the ceaseless neural discharges creating our ruminating thoughts” (43). Notice, already, that this simple point begins to put pressure on the idea that we *describe* pain (or other “inner states”) in the first place. If the real mark of the “inner realm” is constant flux, then the very idea of a “theater of the mind,” that simply “represents” stimuli like wax takes an imprint is absurd [cf. (33)]. The brain is not idle; it's rather simply too active for this, constantly discriminating and categorizing, constantly working, predicting, even as we daydream.

Pain has long been known to be dependent on cognitive and psychological contexts (44). Anecdotal evidence strongly suggests that severe wounds are often ignored during battle, whereas longer waiting times at the dentist’s office heighten the pain of a needle prick. Presentation of a sensory stimulus during periods of heightened default-mode activity weakens our perception of that stimulus (45). Rhythmic brain activity in the form of oscillations immediately preceding the presentation of auditory and visual stimuli has been known to bias the sensory perception of such stimuli (46–50). In other words, the state (i.e., brain state) a person is in at the time of a noxious event determines the psychophysical pain outcome. Indeed, more boldly, the brain's antecedent processes are actually simulating (i.e., predicting) and shaping the experience itself. These considerations recast pain not as a static “given” but a dynamic “construction.” In turn, this begins to put pressure on the representationalist claim that discreet neural correlates represent noxious stimuli and are sufficient for pain experience [e.g., (51) for a further push in this direction]. Simply, there is no evidence for such stable correlates or common neural references for pain experiences. Moreover, such a dynamic account facilitates a very different way of conceptualizing how noxious stimuli, nociceptive and neural activity, and pain experiences align. **Instead of looking for some sort of stable link between stimulus, neural activity, and experience, a more fruitful approach may be listening for Bachian counterpoints where different “melodies” come into a global “harmony” of pain**. Interestingly, such an account seamlessly harmonizes (pun intended) with recent work that seeks to bridge the gap between phenomenological investigations into the illness of pain, i.e., how pain manifests certain invariances in experience as it develops, and the neuroscientific (and broader objective) research into pain as a disease whose neurophysiology evolves and ramifies over time [e.g., (52)].

Our sensory experiences, including pain, are critically dependent on coordinated and mutually interrelated communications between the S1 cortex and thalamus (53) (see previous paragraph on Cartesian dualism). Various pioneering theoretical models of the thalamocortical pathway have been proposed, including the physiological basis of thalamic alpha rhythms by Andersen and Andersson in the late 1960s, which builds upon earlier investigations in the 1930s by Bremer, Morison, and Moruzzi [reviewed by (53)]; the seminal hypothesis of thalamic burst firing and thalamocortical dysrhythmia by Llinas [(54), also reviewed by Llinas in (55)]; Steriade and Sherman's debate regarding the significance of thalamic bursts as a “wake up call” (56, 57); Melzack's theory of the pain neuromatrix, a widely distributed brain network that is genetically determined and modified by sensory experience (58); and Craig's framework of the central homeostatic afferent pathway (59). All of these conceptual frameworks influenced our modern neuroscientific understanding of pain in significant ways, some continue to be contested (36, 57), none provided a unifying model that fully explains the representation of pain experiences by neural circuits in the brain, and the dependence of this representation on prior brain states.

Granting this, if “I have a pain” describes something buried in the brain, the anatomically distributed and functionally dynamic neural circuits mediating that pain experience are themselves dependent on an active discourse between internal and external processes, as well as priors and expectations, as we will further discuss below, hence that something cannot be entirely private. However, the pain experience is fundamentally subjective in the sense that it depends on a person's evaluation of her current state. Let us develop this further. According to a recent hypothetical framework, a consciously alert brain is constantly engaged in *predictive coding* to anticipate the future (60, 61). Reaffirming deficiencies in the Cartesian view, Saab and Barrett proposed the embodied predictive interoception coding (EPIC) model, an active inference model that anticipates, rather than reacts to, external stimuli, whereby brain state arguably plays a critical role in pain perception (62). According to EPIC, The flow of information in the “resting” brain travels from higher areas in the processing hierarchy (agranular cortex, e.g., limbic and motor areas) toward lower areas (granular cortex, e.g., S1). The difference between predictions and sensory input (“prediction error”) is sent back up the hierarchy via the thalamic reticular nucleus, evident in thalamic burst firing patterns. Indeed, EPIC has been attributed to a wide range of cognitive and affective illnesses (60).

Accepting EPIC for a moment, several crucial interdependent points follow:

(1) The experience of pain does *not* flow in a Cartesian, unidirectional manner from, e.g., tissue damage or *Lovecraftian* ghosts that suddenly haunt the theater of my mind into conscious experiences and then onto languages. Instead, recognizing pain depends on the brain actively anticipating that some stimuli or signals have a high likelihood of being painful, based on prior experiences, present context, and future consequences.
(2) Such predictive coding can be harmonized with a broad Bayesian account of updating. As it were, the brain registers something as a pain not only based on brute occurrent noci-perception but also on expectations, derived from the past. As it were, the brain goes in “front-loaded” with a prior “bet” concerning how likely some given factor is to be pain and conditionalizes this new factor against this background.
(3) Given this, some understanding of the prior probabilities of pain recognition is required. Moreover, such an understanding has to be conceptually independent of sensations, for the math to work. And here, a vital point is that the Bayesian framework places very few strictures on what my prior probabilities are, where I get them from, etc. More specifically for this context, Bayes’ theorem in no way restricts the priors to what goes on in my skull.
(4) Pursuant to this, one feature that Bayesians emphasize is that these prior probabilities do *not* track objective features of the world like resonant frequencies but reflect my own evaluations of how sure I am that some event will occur (or has just occurred). Moreover, subjective Bayesians insist that any prior distribution that respects the basic axioms of probability theory is coherent and rational. Given this, **a Bayesian framework can capture certain features of the subjectivity of pain without falling prey to the privacy trap**. Specifically, pain is so person-relative exactly because different individuals may have different priors when they experience the same sensation, leading to different posterior distributions. Indeed, Bayesian pain researchers utilize variations in priors to partly explain why some people develop chronic pain conditions and others do not (63). In addition, the relevant expert who has access to the most informative priors concerning pain recognition is the person herself. Intuitively, different people make different “bets” about what features will provoke pain, the person who made the “bet” is in the best position to know how much she “bet,” and these “bets” change what sensations end up being recognized as painful. Hence, pain is subjective in the sense that it is *personal*, i.e., it depends on the person's priors and she is the relevant expert. Importantly, the dynamic flux in brain states ensures that an individual's sensory experience is always unique, even in response to the same stimulus. However, none of this is because of eldritch terrors that haunt my mind or hidden neuro-states buried too deeply in my brain. Instead, it is a simple consequence of Bayes's subjective view of probability. It is math, not magic, that partly explains pain's subjectivity, without falling into the privacy trap.
(5) In turn, this implies that we need some account of where different people pick up their varied priors. And this cannot bottom out in Bayesian updating *per se*. This is because such a view triggers a regress as each prior is itself the result of a past conditionalization *ad infinitum* [cf. (64)]. And, although the question of how we get our ur-priors has not been given the attention it deserves, Patrick Suppes offers a pathbreaking account [e.g., (65)]. At base, Suppes makes three key points. One, explicit ur-priors are the end result of a long process of learning. Two, this learning can be captured in terms of associations wherein certain constantly conjoined features in experience form and strengthen various conceptual connections. Three, these connections depend on largely tacit know-how that changes the shape of the experience itself. In this, Suppes seems to agree with Wittgenstein's provocative remark that experience rests on techniques. More formally, the similarities and differences I attend to over the course of an experience presuppose relevancy criteria that turn on a specific form of training that highlights certain similarities/differences as relevant and discounts others. Or, more intuitively, my training shapes how I experience and so the lessons I can draw from it. For example, consider looking at an electroencephalography (EEG) readout. One of us (CD) sees in the readout a set of arabesque waves that may be quite beautiful but have no information content. In marked contrast, the other (CS) sees the brain's electrical activity. Critically, this difference is *not* in the readout, but *how* it is experienced. And this seeing rests on neuroscientific training or a sad lack of it, as the case may be. *Notice that this seamlessly integrates with the above discussion of brain states. The brain does “not” passively receive information from the “inner world,” but actively shapes it*.
(6) Granting that Suppes is right, it turns out that both my body's reactions to noxious stimuli and my community's response to pain expressions *cannot* be discounted as mere accidents. For the body, the fact that certain reflexive reactions to stimuli causally antedate my experience of pain readily lends itself to associative learning. For example, I stick my hand in a fire, it reflexively withdraws, and *then* I experience pain. And these elements become conjoined in a reflex arch wherein I pick out certain stimuli as potentially painful based on my past reactions and my reactions to certain stimuli determine that they are painful. In turn, these linkages can become explicated in explicit prior probabilities, e.g., I expect to a very high degree that my hand encountering fire will be recognized as pain. This suggests that my reactions to noxious stimuli may actually form and strengthen associations that terminate in the priors I need to start assessing if new signals are recognized as painful. Indeed, since we need priors and cannot get them by more updating, such associative learning may be essential for pain recognition.
(7) For my community, things are even more interesting, though admittedly more speculative on our part. Let us begin by assuming that human beings are inherently social in that they make sense of themselves in part via the reactions of others to them [for an extremely sophisticated account, see (66)]. I see a joke as offensive, understand a rapid heart rate as lust or fear, etc., based in part on how others react and interact with me. Next, consider that evidence from pediatrics suggests that children struggle with pain recognition and communicating their pain to others, partly because they may be unable to disentangle which aspects of the “inner” hurley-burley are relevant. For example, it seems like children are unable to distinguish between pain and broader distressing symptoms, though, admittedly, the evidence is hard to fully interpret [e.g., (67)]. In any case, a qualitative study of 3- to 6-year-old boys post-operatively failed to distinguish between pain, nausea, fear, and anxiety, often using “pain” (and other cognates) as a catch-all for feeling unwell (68). Relatedly, a quantitative study of older children (9–18 years old) found that pain-ratings and negative affect ratings were so strongly correlated that distinguishing between them may be well neigh impossible at the moment of assessment, suggesting things are likely even worse for younger children (69). Moreover, one of the few systematic reviews on pain scales for 3- to 4-year-old children found that most seem incapable of using the tools in a valid and reliable way for pain intensity (70). Tellingly, many young children seem to rely on emotional co-regulation, i.e., checking their parents’ reactions, to grasp how much distress they (should?) have (71). More interestingly still, a study on pain-language acquisition emphasized that young children often use pain terms to elicit attention and care from their parents (72). Together, these findings suggest a very interesting, and deeply Wittgensteinian, thought. To wit, pain-talk for young children is *not* simply a verbal representation of the “inner” world. Instead, it is more of a request for help. In turn, this may mean that a child's tendency to conflate pain-talk with bad feelings generally is far more akin to how children cry when they are hurt, scared, nauseated, etc. than it is to an informative utterances about how things are. And, if this is so, it may be that certain responses of others help formulate the relevancy criteria that allow the child to distinguish which signals are relevant and which to ignore. For example, when a mother responds to the eruption of chaos that follows from a child's fall by kissing the scrapped knee, she may well be providing the child with relevancy criteria that determine which aspects should be attended to (e.g., the scrap, the ache) and which aspects should be ignored (e.g., the rapid heart rate, the fear). Moreover, as noted above, it may be that the extension of the set of experiences that a person counts as pain is partly determined by normative response patterns others have to them. Flowing from this, and more interesting still, is that *how* “pain” is used in various communities may itself change the prior probabilities of pain recognition by shifting the relevant aspects that are recognized in the first place. And, wild as it sounds, this may capture important variations at work in the experience of pain. To see this, consider couvade rituals that occur in various cultures [e.g., (73)]. These rituals seem to enable a pregnant female's male mate to ascribe and self-report various symptoms, including pain, as a direct result of the female's gestation. And it may be that this culturally specific use of “pain” in such rituals feeds into the prior probabilities of pain recognition for these males by changing certain associative connections. Needless to say, one can multiply such examples–mystics who feel Christ's pain, moms who feel their child's ache, etc. Stepping back from such potentially controversial cases, evidence *does* suggest that pain recognition is mediated by social contexts, as systematic reviews make clear (23). And a broadly Bayesian account easily accommodates this by claiming that shifts in context may shift priors and so change posts. Finally, and even more radically, this can go *both ways*. In some contexts, I *do* feel the pain of another, exactly by shifting my priors. Hence, as Wittgenstein said, pain enters the language-game, but *not* as a picture.
(8) This Bayesian account can be modified to accommodate pain-talk in a way that is much more clinically fruitful than the thought that someone making a pain-report is, per impossible, describing their pain to me. To see this, let us assume that pain-talk *testifies* to the presence of pain in another. And let us further assume that I assign to this testimony a certain degree of credence, based partly on how empathetic I am towards them. Notice that, unlike a descriptive account, this credence account is nicely supported by the neuroscientific literature that shows that empathy for another person's pain is crucial for how people recognize it. Comparing brain activity in individuals experiencing painful stimuli to that elicited in a loved one present in the same room, a distinct neural substrate for empathic experience emerges (74). In mice, the transfer of pain through social contact has been demonstrated to be mediated by neurons in the anterior cingulate cortex that are common with neural circuits for the human empathic experience (75). Moreover, such an account also aligns with the phenomenological insight that human behavior is “always already” expressive [e.g., (76)]. For example, I do *not* first observe that lacrimal ducts are secreting saline with some additions, and then *infer* that the child is in pain. Instead, I *see* that they are crying *in* pain. Here, we see another striking harmony between neuroscience and phenomenology (ibid). We *see* the child as *crying* because the very act of perception may “always already” mobilize the neural substates associated with empathy. Indeed, to borrow from an old science fiction movie, imagine *how hard* it would be to explain to a robot why people secrete saline from their faces if they do not already see this as a sign of pain. And, again, this suggests that I *can* experience *another's* pain, and that pain-talk can help mediate this. Moreover, it also gives us powerful tools to isolate and correct epistemic injustices. For example, a provider might assign high credence to a prior (and utterly false!) hypothesis that African Americans are less pain-sensitive than whites. Or she might not form an empathetic relationship with, and so assign low credence to, the testimony of a “white working class” person she deems to be “drug-seeking.” In either case, the provider's recognition of pain will fail, and inequities will result. And, in both, using Bayesian epistemology to precisely identify where such errors enter gives us ground to correct them. Needless to say, this all promises to be far more fruitful than making literal non-sense of pain reports.

In closing, notice that this vindicates the idea that the ground truth of pain is a subjective narrative while dropping the privacy baggage. Pain is subjective for a myriad of reasons including that it depends on the person, that the person's priors may well be unique to her, that these priors are formed and strengthened by her unique life history, culture, particular neurophysiology, etc. Moreover, given that pain is a complex experience, it is extremely likely that the factors that generate, sustain, and modulate pain are dynamically interacting and may well be in nonlinear relationships with each other, implying that we may simply never develop a totally tractable way to capture it formally. This is all for the good. However, *none* of this entails that there are spooks that haunt my mind or neuro-alchemic procedures that let machines create experiences. And *none* of it means that the project of expanding pain assessment to include biomarkers, fMRI scans, etc., is somehow flawed. If anything, we need *more* and *more varied* sources of information, not (failed!) drastic reductions of multidimensional and multimodal experiences to numerals we do not understand (be these patient reports or “pain meter” read-outs). Hence, pain *is* subjective– let us begin taking this truism seriously!

### 1.9 Summarizing arguments against pain privacy and objective pain diagnostics

Pain is far more complex than overly simplified feedforward Cartesian machine models or unidimensional experiential Cartesian ghost concepts can handle, be these in neuroscience or philosophy of mind. And this is for the good as pain is, in fact, multimodal, pain-talk is polysemic, various social and cultural factors change pain recognition, and talking with patients about pain shows how multifarious and ramifying it is for them.

Irrespective of the underlying mechanisms giving rise to the spontaneous ebb and flow in brain activity and the different labels attributed to *brain state* (default mode, endogenous state, etc.), ongoing fluctuations in brain activity definitely bias our perception of external events. This stands in stark contrast to the antiquated view of the reflex arc as a fundamental model of the brain (i.e., the flow of electrochemical signals relaying information about a noxious stimulus from peripheral nerves to the cortex where pain suddenly emerges as an epiphenomenal response to a biological process). However, *brain state* which includes ruminating thoughts, past events and expectations, is one of the most frequently overlooked determinants of pain perception. Pain is mediated by dynamic, interrelated communications in the thalamocortical network, whereby perceived noxious stimuli are modulated by ascending thalamic drive, as well as top-down feedback from cortical areas that mediate interoceptive information, memories, future predictions, and information about events in the external environment. This rich interplay between inner and outer variables that ultimately shape the pain experience argues against pain being accidental to biology, rather **pain is a deliberate process of making sense out of a noxious stimulus through a neural process that pools information from multiple sources in the brain and broader community**, a process whereby the individual invites others to become active participants in the experience.

In sum, pain is subjective because it is *personal*. In other words, what a person recognizes as pain turns partly on her priors, and these depend inexorable on the complex web of her life history, cultural environment, religious convictions, genetic endowment, neurophysiological variations, etc. However, none of this entails that pain is *private*, any more than a highly specific nutrition plan from a dietitian that tries to take into account a range of metabolic, cultural, spiritual, genetic, etc., factors is private. And, provided this is right, the walk-away lesson is felicitous. Pain's subjectivity requires *personalized* medicine, not unworkable objective meters or incomprehensible private languages.

## 2 Showing the fly the way out of the fly bottle: implications of refuting pain privacy for healthcare

Communicating our subjective pain to others and the appreciation of our pain by others, defies the logic of objective diagnostics and privacy. This principle constitutes a cornerstone of medicine and the caring for others. The alternative that my pain can be measured irrespective of my narrative or that I cannot communicate my private pain to others because they cannot have my experience, is counterintuitive, a dead-end in medicine and a devastating outcome for society. So, if pain cannot be measured objectively and yet be communicable, how can pain be assessed *comprehensively*, and what kind of tools and methods can we develop to capture its multiple dimensions? What is left with no ghosts and no machines?

As we hope we have made clear, one key project is expanding the data set we consider when assessing and treating pain. And part of this expansion, as the NIH's guidance on pain biomarkers suggests (41), is focused on behavioral, genomic, and electrophysiological biomarkers, as well as general-purpose digital health biomarkers in the form of wearables devices, collectively referred to as *modalities*. Although there is no general consensus on the approach to developing such biomarkers, a common theme seems to be the detection of signs and symptoms that *correlate* with pain, e.g., autonomic responses including sweat and pupillary reflexes, expression of specific genes that predispose individuals to chronic pain, circulating inflammatory cells in blood, neural activation in the brain, etc. Such research promises to be extremely fruitful if used properly. Indeed, pairing a subjective report of pain with objective correlates that, e.g., clarify what sort of pathophysiological process is afoot can guide clinicians’ selection of treatment modalities. Moreover, we would stress that biomarker research should be carried out beyond the body and outside the brain. By this, we mean that careful ethnographic, sociological, and epidemiological research into what factors modulate pain, how they do so, and where they vary would provide us with an even richer set of data to consider. Finally, we wholeheartedly support using better bedside clinical assessment tools. For example, the Defense and Veterans Pain Rating scale, a scale adopted in the early 2010s by the US Department of Veterans Affairs and that integrates functionality into all pain assessments, strikes us as a laudable next step in pain scales because it explicitly includes data about functionality with testimony. Moreover, it would be extremely useful for biostatisticians to develop and validate structured questionnaires that go beyond the “sharp? Leg? 7? Four-day duration?” sort of questions. In sum, once we get over the either privacy or objectivity dichotomy, once we realize that the ground truth of the patient's narrative can be supplemented by sources of information that clinicians and researchers can identify, we see an ocean of data before us that needs to be explored and that we firmly believe will refine our ability to alleviate suffering.

That said, because pain is multidimensional, **it is unlikely that a single modality will succeed as a stand-alone biomarker**, especially not without incorporating the subjective elements as ground truth of the pain experience. And at present, there are no known biomarkers for the subjective factors that shape an individual's pain experience, such as memory, present context, and expectations. Since a comprehensive approach to assessing pain is necessary, researchers are beginning to acknowledge the challenge of ingesting and analyzing large amounts of multimodal data using artificial intelligence (AI) and machine learning. With the current buzz around AI, it has become tempting to envision algorithms that *automate* pain assessment, essentially taking the patient out of the loop. Obviously, this contradicts our thesis which argues that pain is neither purely objective nor wholly private, but intersubjective, i.e., it is something that we interpret by considering how others react to us. Worse, if our account is correct, then replacing pain assessment with AI may well worsen the experience for the patient. And, this is exactly because a patient's expressions of pain, and the sympathetic reactions of caring providers to it, may modulate the actual experience of it. Indeed, talking about pain may help alleviate it. More formally, if we are right, then the intersubjective recognition of pain may change the associative connections in priors and this shift will alter the posterior probability, thereby altering what is recognized as pain. Suffice it to say, it seems unlikely that AI-generated algorithms can do this because, if for no other reason, people want sympathy from people, not programs. Happily, though, this in no way means that using AI and machine learning is in itself useless. Tools, data, patterns, etc., are all vital. The mistake is asking them to do what they cannot.

Finally, and provided that this is on the right track, the EPIC Bayesian model may lend itself to other areas of medicine as well. For example, it seems to nicely harmonize with ongoing Bayesian research into the positive symptoms of schizophrenia. Additionally, it may help explain sociogenic illnesses, like the increased prevalence of Tourette's symptomatology among young social media users, as a sort of re-jiggering of priors and so a shift in the posts (77). Indeed, we may even begin to overcome the deeply entrenched idea that people are a fusion of ghostly symptoms and measurable signs.

In sum, we are firmly convinced that discovering biomarkers and expanding research in that area generally can help us address and redress pain. The mistake we fear is when researchers come to think these can replace a patient's narrative. This is succinctly summarized in what we have heard from patients and clinicians: “We hate the VAS, but don’t take it away from us!”

## Data Availability

The original contributions presented in the study are included in the article/Supplementary Material; further inquiries can be directed to the corresponding author.
